# A Small Cut, a Big Challenge: Pediatric Mucormycosis in an Unexpected Host

**DOI:** 10.1002/ccr3.70176

**Published:** 2025-02-07

**Authors:** Marriam Tanvir, Ali Tanvir, Saad Khan, Fathimathul Henna, Syeda Mashal Fatima, Aqsa Munir, Javed Iqbal

**Affiliations:** ^1^ Khawaja Muhammad Safdar Medical College Sialkot Punjab Pakistan; ^2^ King Edward Medical University Neela Gumbad Lahore Punjab Pakistan; ^3^ Saidu Medical College Swat Khyber Pakhtunkhwa Pakistan; ^4^ Dubai Medical College for Girls Muhaisna 1 UAE; ^5^ Khyber Medical University Peshawar Khyber Pakhtunkhwa Pakistan; ^6^ Dow Medical College Karachi Sindh Pakistan; ^7^ Nursing Department Communicable Diseases Center Hamad Medical Corporation Doha Qatar

**Keywords:** immunocompetent, Mucormycosis, orbital infection, pediatric, Rhizopus

## Abstract

Mucormycosis is an infrequent fungal infection commonly seen in immunocompromised individuals. However, it can also occur in immunocompetent patients, especially following trauma. We present a case of a 15‐month‐old girl with increasing swelling and redness in her left eye after a minor traumatic injury. Imaging showed sinusitis with orbital involvement. Histopathology validated the diagnosis of mucormycosis caused by Rhizopus species. The patient had emergency surgery, followed by antifungal and antibiotic therapy. Despite febrile reactions to the treatment, the infection was managed, and the patient was discharged after 2 months. This case highlights the importance of considering mucormycosis in immunocompetent patients with unexplained ocular symptoms after trauma. Early diagnosis and aggressive treatment are essential for improving outcomes, especially in pediatric patients.

**Summary**

- This case highlights the critical need for increased awareness of mucormycosis among clinicians, especially in younger patients presenting with unknown ocular symptoms following trauma.
- Early diagnosis through histopathology and imaging is most important.
- Treatment challenges, such as controlling amphotericin B–induced fever and maintaining renal function, demonstrate the importance of close monitoring.
- Ultimately, proper detection and aggressive surgical and antifungal treatments are necessary for improving patient survival outcomes in such complex cases.

## 1 Introduction

Mucormycosis, a devastating fungal infection caused by “*Rhizopus*” species, is predominantly observed in individuals with compromised immune systems, such as those with diabetes, malignancies, or receiving immunosuppressive treatments. Despite being rare, this infection can also emerge in immunocompetent individuals, often triggered by factors like trauma [1]. While it is rare, recognizing mucormycosis in such patients is crucial, as the infection can progress rapidly, leading to significant morbidity and mortality if not promptly treated. Although mucormycosis is primarily linked to immunosuppression, recent studies have shown that trauma can act as a potential gateway for fungal invasion in healthy individuals [2]. This presents a unique diagnostic challenge, particularly in pediatric patients, who may exhibit nonspecific symptoms following minor injuries. The delay in diagnosing mucormycosis in such patients can result in poor outcomes due to the aggressive nature of the infection [2].

Early recognition, accurate diagnosis, and timely intervention are, therefore, essential, especially in cases where trauma serves as the initial trigger in an otherwise healthy host. This case report aims to document a rare instance of mucormycosis in an immunocompetent pediatric patient following minor trauma, focusing on diagnostic challenges and clinical management. By detailing this case, we aim to raise awareness about the ability of mucormycosis to occur in healthy individuals, particularly after trauma, and to underscore the importance of considering fungal infections in the differential diagnosis for pediatric patients presenting with unexplained ocular or sinus symptoms. Additionally, the report seeks to contribute to the growing body of literature on mucormycosis in immunocompetent patients and highlight the need for further research on early detection and management strategies.

## 2 Case History/Examination

**FIGURE 1:**
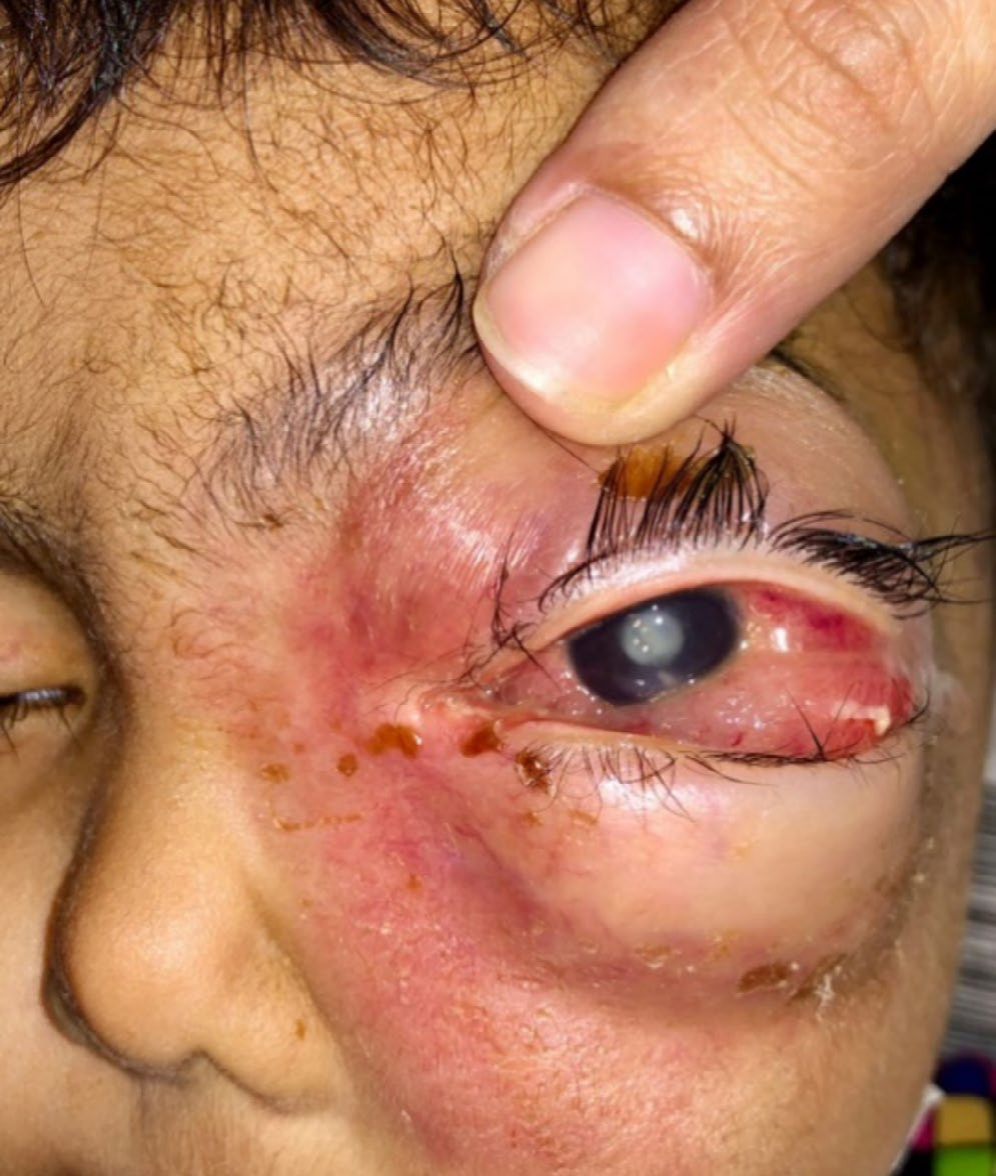
The clinical presentation of the patient's right‐sided periorbital edema, erythema, proptosis, and conjunctival chemosis.

A 15‐month‐old girl was brought to the Pediatrics Emergency Department with complaints of progressive swelling and redness of the left eye for the past 3 weeks following a minor traumatic injury (fall from bed). On physical examination, there was right‐sided periorbital edema with erythema of the skin, proptosis, conjunctival chemosis, and extraocular motility defects, as shown in Figure 1. There was an aversion to covering the right eye and no response to light in the left eye. The right eye appeared normal, with intact reflexes and visual acuity. Nasal per speculum examination revealed no redness or other signs of inflammation in the nasal mucosa. She had no past significant medical history and was born on term via a spontaneous vaginal delivery.

**FIGURE 2:**
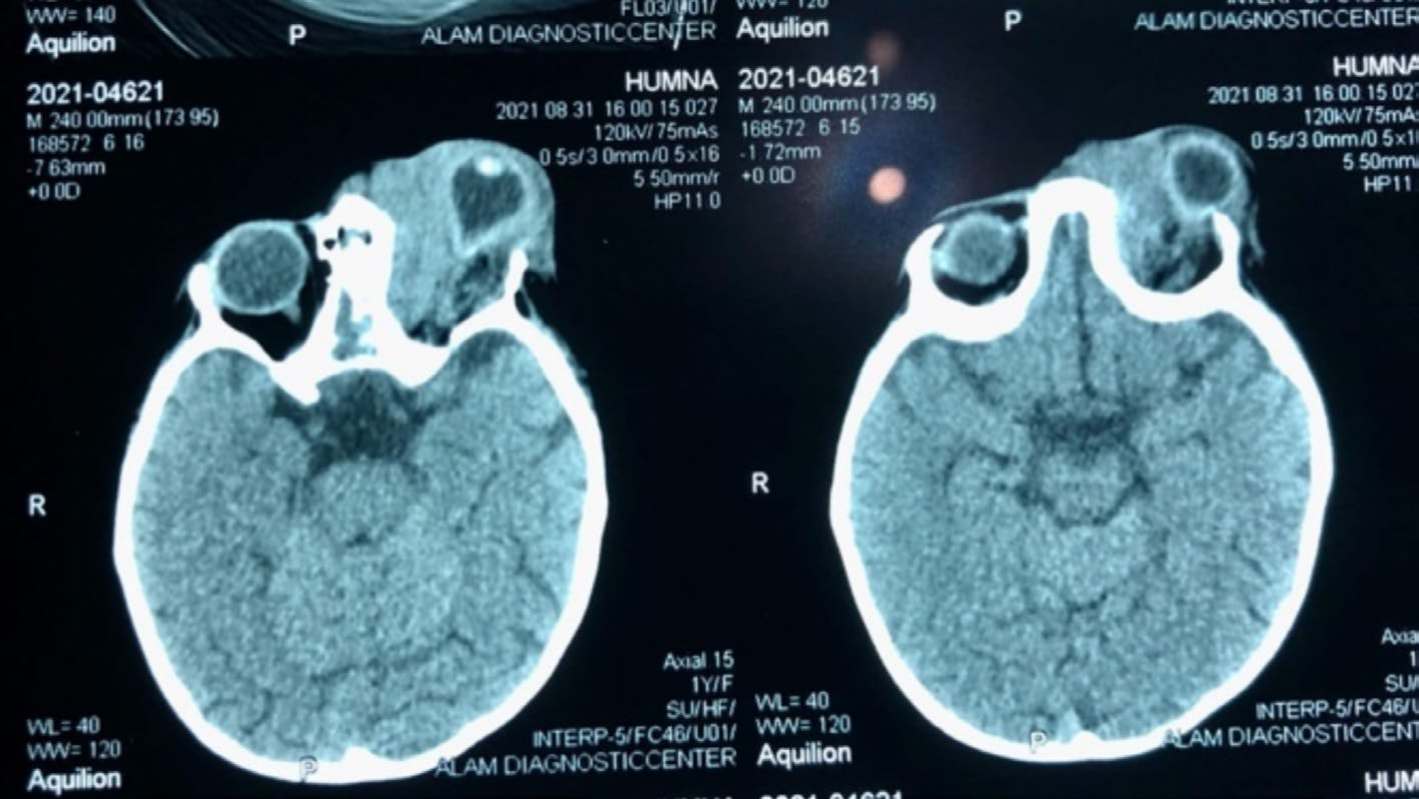
CT scan showing sinusitis involving the right ethmoid and maxillary sinuses, along with the orbital mass.

**FIGURE 3:**
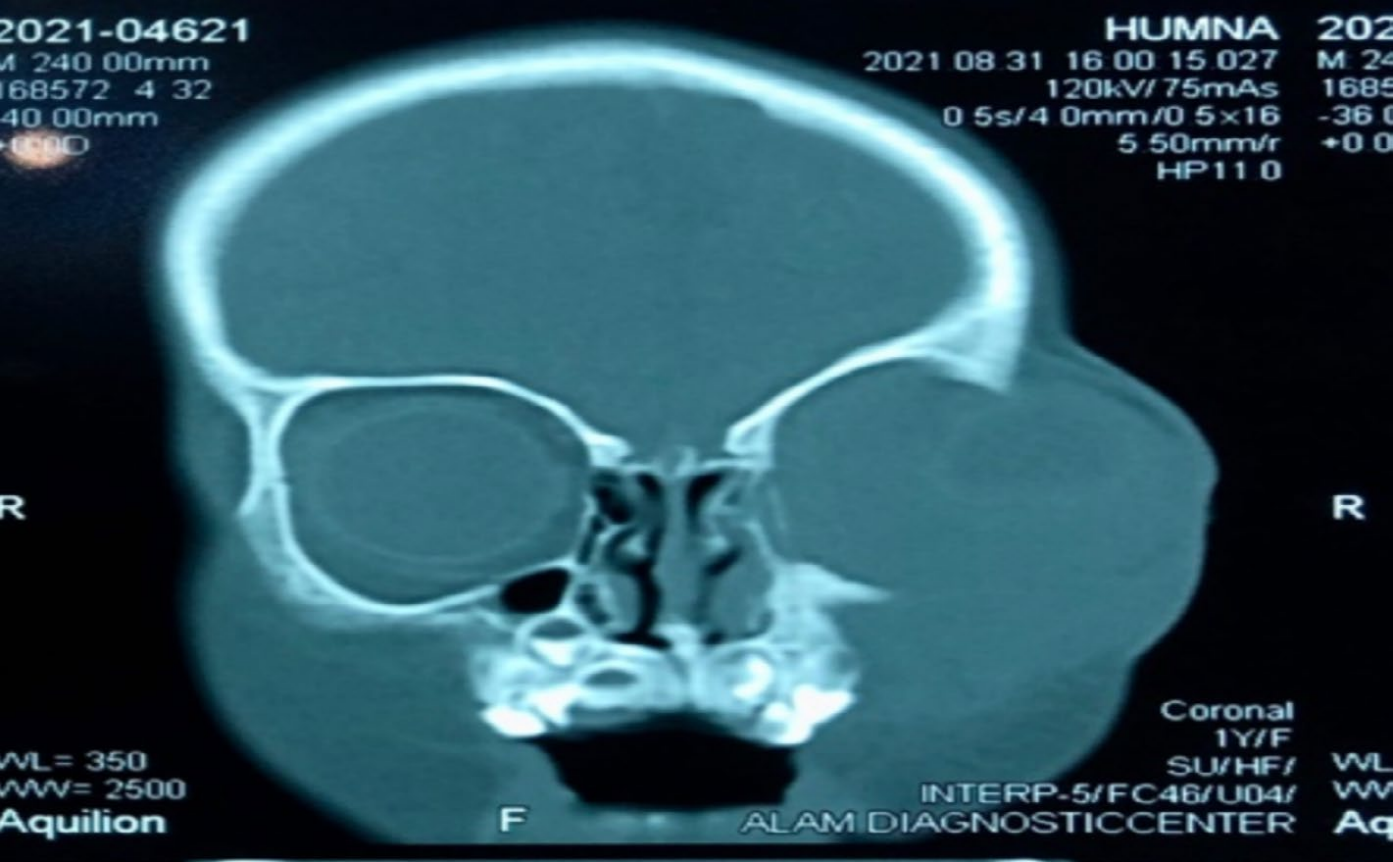
Another view of the CT scan highlighting the osteolysis of the maxillary sinus and orbital floor.

Her lab investigation for a fasting Basal sugar level (BSL) revealed a glucose level of 61 mg/dL negating diabetes (70–9 mg/dL). Complete blood count (CBC) was normal except for an increased total lymphocyte count (TLC count), attesting to a lack of immunosuppression. Her liver function tests (LFTs) and renal function tests (RFTs) were normal. A plain and contrast‐enhanced computed tomography (CT scan) of the brain and paranasal sinuses was performed as shown in Figures 2 and 3. It was suggestive of sinusitis involving the right ethmoid and maxillary sinuses extending into the inferior wall of the right orbit. Orbit showed an ill‐defined mass occupying the inferior and medial orbit, surrounding and compressing the eyeball from all sides; however, no intracranial extension was noticeable. Osteolysis of the lateral and medial walls of the maxillary sinus and orbital floor was noted. Brain parenchyma was normal on CT.

Emergency surgery was performed on the third day of presentation after anesthesia optimization to obtain a biopsy. Through anterior orbitotomy along the inferior orbital margin with a slight extension of the incision medially along the lateral nasal wall for adequate exposure and complete excision, a friable mass (with a fish flesh appearance) was removed and sent for histopathology.

**FIGURE 4:**
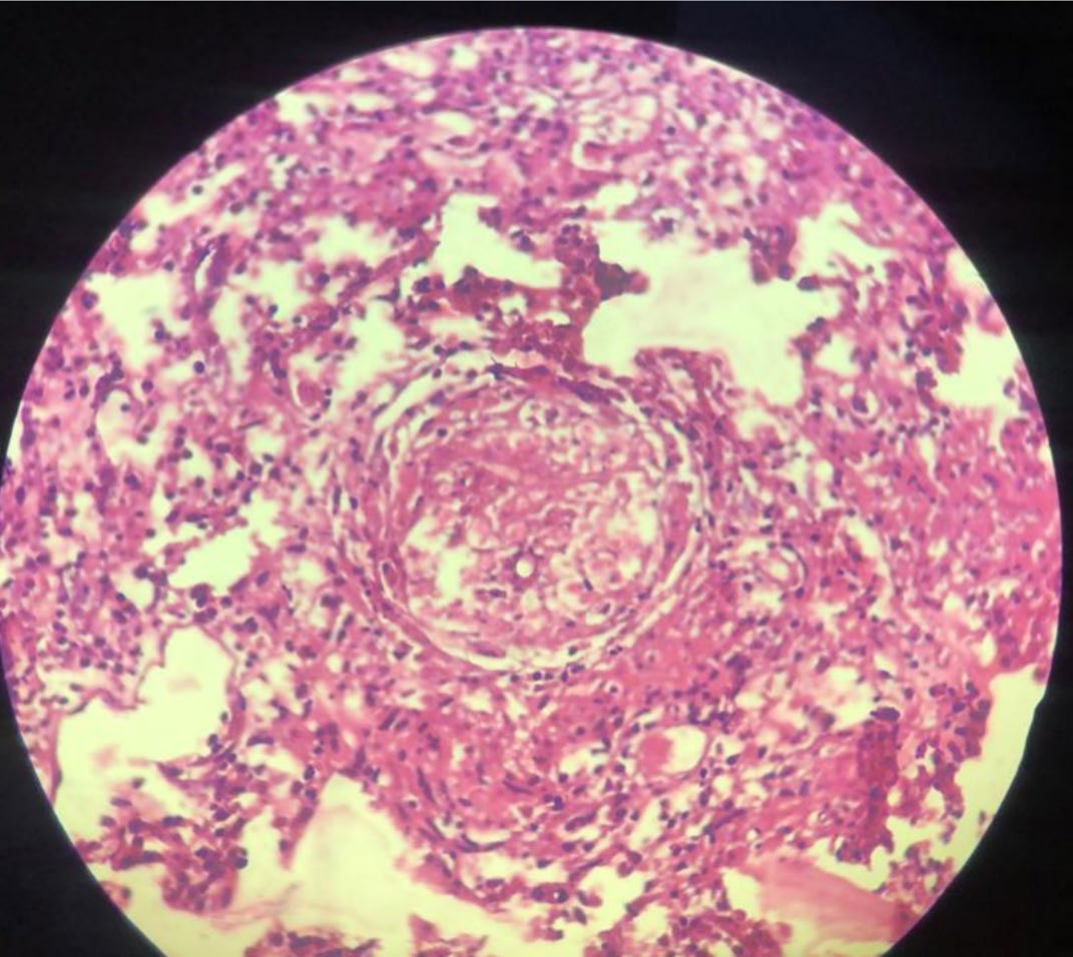
The histopathological examination with hematoxylin and eosin staining, showing broad, nonseptated hyphae indicative of mucormycosis.

The patient was suspected of rhabdomyosarcoma, though subsequent histopathology results suggested infection with *Rhizopus*. Due to a lack of convincing clinical evidence, the pathology department was requested to review the sections (Figure 4). Histopathologic results from tissues retrieved during orbital debridement revealed scattered epithelioid cells and multinucleated giant cells with hematoxylin and eosin staining along with many refractile, broad, nonseptated hyphae further highlighted with periodic acid–Schiff stain, which supported the diagnosis of mucormycosis. A board of three consultant histopathologists tendered the histopathology report. The patient was transferred to the pediatric unit for further care. She was started on intravenous (IV) amphotericin B at a dose of 5 mg every 24 h, with daily renal function tests (RFTs) for the next 2 months. In addition, she received clindamycin 250 mg, Augmentin 150 mg, and meropenem 180 mg, all given every 8 h. These antibiotics were continued for 3 weeks until the swelling and redness decreased by 50%.

At this stage, IV amphotericin B was continued as the primary treatment. Although the patient responded well to the medications overall, she experienced multiple episodes of fever as a reaction to amphotericin B. These febrile episodes were managed with paracetamol infusions and by administering the amphotericin B infusion at a slower rate.

After 2 months of treatment, the patient was discharged with close follow‐up. At discharge, the disease was in remission, but imaging, including ultrasonography, revealed that her affected eyeball had become nonfunctional (phthisical) with scarred and involuted tissue around the eye socket.

## 3 Methods (Differential Diagnosis, Investigation, and Treatment)

The biopsy specimens done during emergency surgery were observed under light microscopy, immunofluorescence, and electron microscopy. On light microscopy, the tissue sections appeared to be extensive necrotizing inflammation involving the orbital tissue, with areas of nonliving tissue and invasive fungal elements. The fungal organisms were seen as broad, nonseptated hyphae with right‐angle branching, compatible with mucoromycetes. There was no proof of granuloma formation. Immunofluorescence staining of the tissue demonstrated negative results for IgG, IgA, C3, and fibrinogen, suggesting the absence of immune complex deposition or vasculitis.

On electron microscopy, the glomerular capillary walls were investigated to opt out vasculopathy or basement membrane association, which were negative. No dense deposits or abnormal fibrillary structures were seen in the tissue. Polymerase chain reaction (PCR) for fungal DNA focusing on the *Rhizopus* species was positive, confirming the diagnosis of mucormycosis. PCR was performed on fresh tissue samples and formalin‐fixed paraffin‐embedded sections. The PCR used primers for the ITS region, and the products were sequenced. The sequence analysis using BLAST confirmed the genus *Rhizopus*.

Tissue samples sent for microbiological culture showed growth of *Rhizopus* species within 48 h. Identification was based on morphological features observed in culture, such as the growth of fast‐growing colonies with characteristic sporangia morphology under microscopy. No biochemical or temperature‐based tests were performed. MALDI‐TOF was not utilized. Antifungal susceptibility testing was done to initiate therapy, and the isolate was observed to be sensitive to amphotericin B. Antifungal susceptibility was determined using broth microdilution, with an MIC value of ≤ 1 μg/mL for amphotericin B. Other antifungal agents were not tested for MIC in this case. CT scans of the brain and orbit appeared to have significant sinusitis with orbital association, including destruction of the ethmoid and maxillary sinuses and invasion into the orbit. There was no known evidence of intracranial extension or abscess formation.

The patient was thoroughly monitored during the antifungal therapy, with daily renal function tests (RFTs) due to amphotericin B toxicity. The patient's progress was tracked with serial imaging, showing gradual resolving of the orbital mass and infection.

## 4 Conclusion and Results (Outcome and Follow‐Up)

This case highlights the clinical importance of identifying mucormycosis as a potential diagnosis even in immunocompetent pediatric patients who underwent minor trauma. Prompt identification and intervention, including surgical debridement and antifungal treatments, are important for improving survival and outcomes. The case also demonstrates the aggressive pattern of the infection and the need for swift and multidisciplinary team management. Increasing awareness about mucormycosis in normal healthy individuals can help reduce diagnostic delays and lead to effective treatment approaches, which will improve patient prognoses. Further research into prevention and management in immunocompetent patients will be necessary to advance the understanding and management of this rare but severe infection.

## 5 Discussion

Mucormycosis is an aggressive fungal infection, often associated with significant morbidity and mortality. This case is unusual as the patient had no predisposing factors like diabetes or immunosuppression. Trauma is an infrequent trigger for mucormycosis, but it likely provided an entry point for the Rhizopus species in this case. This case demonstrates that early surgical intervention and antifungal therapy are crucial for favorable outcomes. This report reinforces the need for clinicians to consider mucormycosis even in immunocompetent pediatric patients presenting with unexplained ocular symptoms posttrauma.

This case is exceptional in that there are no predisposing factors, highlighting the fact that even immunocompetent people can get this potentially fatal infection after a minor injury. A similar case was reported in another study in which a 31‐year‐old male underwent dental surgery, developed necrotizing lesions, and was later on diagnosed with mucormycosis [3]. The dental profession is responsible for being aware of the possibility of potentially severe and potentially fatal complications in healthy individuals [4]. The use of corticosteroids is also a risk factor for mucormycosis. The high death rate, even with improvements in diagnoses, emphasizes the necessity of greater awareness and prompt action in at‐risk groups [5]. In healthy hosts, neutrophils that destroy hyphal components by oxidative burst and macrophages that prevent spore germination are the defensive mechanisms. Infections in diabetics are caused by macrophage dysfunction [6]. Another study shows that GI mucormycosis can occur in malnourished and immunocompetent patients, so if malnourished patients exhibit abdominal symptoms and risk factors, clinicians should consider GI mucormycosis [7]. In this case, the symptoms of the patient were suggestive of rhabdomyosarcoma. The diagnosis was confirmed on histopathology. A study says that in immunocompetent patients, nonspecific symptoms make diagnosis difficult; biopsy and culture are required [1]. In immunocompromised patients with liver cirrhosis, posaconazole can be used safely and effectively to treat mucormycosis [8].

In immunocompetent patients with nonspecific symptoms like this case, essential characteristics for differentiating fungal agents of IFRS include vascular invasion, level of necrosis, pigments, and spores, and the technique used is histopathology [9]. Also, cutting‐edge imaging methods like improved MR angiography and CT angiography offer vital information about the degree of vascular involvement [10]. Allogeneic hematopoietic stem cell transplants, solid organ transplants, and hematologic malignancies are the most common risk factors for pulmonary mucormycosis in which serum quantitative PCR is effective in early diagnosis, especially in neutropenic patients [11]. Managing the underlying disease with early detection and active surgical and antifungal action is often the most important factor for patient survival [4].

More study is required to investigate the epidemiology of mucormycosis in immunocompetent children and determine the best course of action for instances of this nature. To prevent these serious infections, it might also be helpful to look into possible preventative strategies or recommendations for treating minor wounds in children. Research on the long‐term consequences of mucormycosis survivors might also be beneficial in determining their quality of life and recovery paths after infection.

Their receptor activation distinguishes Rhino‐orbito‐cerebral (ROC) and pulmonary mucormycosis: integrin β1 in alveolar cells for pulmonary infections and GRP78 in nasal epithelial cells for ROC. Ongoing research is essential to enhance treatment strategies, focusing on combined therapies and novel antifungal agents due to the persistent high mortality associated with these infections [12].

## Author Contributions

**Marriam Tanvir:** conceptualization, writing – original draft. **Ali Tanvir:** methodology, writing – original draft. **Saad Khan:** writing – original draft, writing – review and editing. **Fathimathul Henna:** writing – original draft, writing – review and editing. **Syeda Mashal Fatima:** writing – original draft, writing – review and editing. **Aqsa Munir:** writing – review and editing. **Javed Iqbal:** writing – review and editing.

## Ethics Statement

Ethical approval does not imply case reports from institutional IRB.

## Consent

Written informed consent was obtained from the patient to publish this report in accordance with the journal's patient consent policy.

## Conflicts of Interest

The authors declare no conflicts of interest.

## Data Availability

As the submitted article is a letter to the editor, there is no additional saved data available. All the data have been extracted from the cited articles.
